# Hemostatic devices in abdominal surgery

**DOI:** 10.1186/1471-2318-11-S1-A10

**Published:** 2011-08-24

**Authors:** F Di Capua, M Petrocelli, M M Saracco, A Vernillo, A Renda

**Affiliations:** 1 Department of Surgical and Anestesiological and Reanimative Sciences, University of Federico II, Naples, Italy

## Background

Bleeding control is an important aim in surgery. A lot of haemostatic devices can be used to support hemostasis, so operative risks and length and hospital stay are reduced.

At General and Transplants Surgery Department of “Federico II” University of Naples, we evaluated the efficacy of hemostatic devices such as fibrin glues and collagen sponges in several surgical applications: kidney, gastroenteral, liver and pancreatic surgery.

Our aim is to find a specific use of each of the devices we used and to identify guidelines.

## Materials and methods

From January 2010 and November 2010, we selected 30 patients older than 70 undergoing major surgery (16 cholectomies, 4 gastrectomies, 4 liver resections, 4 nephrectomies, 2 pancreatic resections); during surgery we used biomaterials.

We examined: amount and nature of abdominal drains, haemocromo and ultrasound control of possible presence of intra-abdominal collections.

We made a retrospective analysis of 30 age-matched patients undergoing surgery from 2000 to 2005, without the use of hemostatic agents.

## Results

We found a shorter operating time and a lower incidence of postoperative complications such as bleeding, lymphocele, biliomi and pancreatic fistulas, of about 15%. In addition, the incidence of complications due to the device itself was low: only one seroma and one case of granulomatous and fibrotic reaction.

## Discussion

The experience of several authors in literature highlights the benefits of biomaterials haemostasis. The results of our experience are in line with those assessments.

## Conclusions

Although you cannot disregard a careful surgical technique and classic methods, larger series and a longer follow-up are needed, the use of hemostatic aids have reduced the operative time and postoperative hospital stay presenting negligible complications and can therefore be considered a valid support.
